# Function and Mechanism of RGD in Bone and Cartilage Tissue Engineering

**DOI:** 10.3389/fbioe.2021.773636

**Published:** 2021-12-15

**Authors:** Meng Yang, Zheng-Chu Zhang, Yan Liu, You-Rong Chen, Rong-Hui Deng, Zi-Ning Zhang, Jia-Kuo Yu, Fu-Zhen Yuan

**Affiliations:** ^1^ Sports Medicine Department, Beijing Key Laboratory of Sports Injuries, Peking University Third Hospital, Beijing, China; ^2^ Institute of Sports Medicine of Peking University, Beijing, China; ^3^ School of Clinical Medicine, Weifang Medical University, Weifang, China; ^4^ Beijing National Laboratory for Molecular Sciences, Center for Soft Matter Science and Engineering, Key Laboratory of Polymer Chemistry and Physics of Ministry of Education, College of Chemistry and Molecular Engineering, Peking University, Beijing, China

**Keywords:** (adhesion peptide) RGD, Arg-Gly-Asp, bone, cartilage, tissue engineering

## Abstract

Bone and cartilage injury is common, tissue engineered scaffolds are potential means to repair. Because most of the scaffold materials used in bone and cartilage tissue engineering are bio-inert, it is necessary to increase the cellular adhesion ability of during tissue engineering reconstruction. The Arginine - Glycine - Aspartic acid (Arg-Gly-Asp, RGD) peptide family is considered as a specific recognition site for the integrin receptors. Integrin receptors are key regulators of cell-cell and cell-extracellular microenvironment communication. Therefore, the RGD polypeptide families are considered as suitable candidates for treatment of a variety of diseases and for the regeneration of various tissues and organs. Many scaffold material for tissue engineering and has been approved by US Food and Drug Administration (FDA) for human using. The application of RGD peptides in bone and cartilage tissue engineering was reported seldom. Only a few reviews have summarized the applications of RGD peptide with alloy, bone cements, and PCL in bone tissue engineering. Herein, we summarize the application progress of RGD in bone and cartilage tissue engineering, discuss the effects of structure, sequence, concentration, mechanical stimulation, physicochemical stimulation, and time stimulation of RGD peptide on cells differentiation, and introduce the mechanism of RGD peptide through integrin in the field of bone and cartilage tissue engineering.

## Introduction

Bone and cartilage injuries are common and frequent (Deng et al., 2019), and mature articular cartilage is limited in its ability to repair itself (Krishnan and Grodzinsky, 2018). eventually lead to osteoarthritis, which causes joint pain (Barnett, 2018). Recently, scaffolds with composed with seed cells became a promising method for bone and cartilage repair (Daly et al., 2017; Shadjou et al., 2018; Zhang et al., 2020). Seed cells are key to tissue engineering, as autologous cartilage and huge bone defect lacks the ability to regenerate, and seed cells could enhance tissue repair by producing extracellular matrix (ECM) and growth factors (Zhang et al., 2016). Scaffolds not only have basic functions, such as supporting and filling, but also promote cell adhesion, proliferation, and differentiation.

In recent years, the use of tissue engineering scaffolds to repair bone and cartilage damage developed quickly. According to the source of scaffolds, they can be divided into natural materials and synthetic materials. Natural materials used for bone and cartilage repair include collagen, hyaluronic acid, fibrin glue, chitosan, agarose and alginic acid. They have good biocompatibility, cell adhesion, and degradation products are non-toxic physiological products (Wang et al., 2021). However, they also have many disadvantages, such as: limited source, difficult processing, poor mechanical strength, and possible disease transmission problems (Rahimi et al., 2021). To solve these problems, researchers have made many attempts in synthetic materials. Synthetic materials commonly used in bone and cartilage tissue engineering include alloys, bone cements, PEG polymers, and poly (ε-caprolactone) (PCL) (Jiang et al., 2021). Synthetic materials indeed solve the problems of natural materials, but they usually have limited cellular adhesion properties.

Cell adhesion is an important condition for long-term survival of transplanted cells (Lee et al., 2015). Due to the bio-inert of most synthetic materials, cell adhesion peptide RGD is usually integrated into biomaterials to achieve better cell adhesion. RGD combined with PCL (Richbourg et al., 2019; Alipour et al., 2020), titanium alloy (Dard et al., 2000; Alipour et al., 2020), and calcium phosphate cements (CPCs) (Lin et al., 2019) had been reported. Many scaffolds for tissue engineering have been approved the possibility to be used in clinic due to high-water absorption ability mimicking natural tissues, easy precision regulation, and low immunogenicity (Chin et al., 2018; Di Palma et al., 2021). PEG is also bio-inert and is often combined with RGD for tissue engineering repair.

It is well known that RGD works through Integrin. RGD has been widely recognized as a polypeptide that enhances cell adhesion and cell viability, its effect on cell differentiation is highly controversial (Burdick and Anseth, 2002; Benoit and Anseth, 2005; Yang et al., 2005; Vonwil et al., 2010; Jäger et al., 2013; Callahan et al., 2013b; Kim et al., 2015). Integrins are a superfamily of cell-adhesion receptors that bind to cell surface ligands (Takada et al., 2007), is a transmembrane receptor composed of α and β subunits, which is closely involved in many important physiological activities of cells, such as cell proliferation (Marsico et al., 2018), cell adhesion (Ellis and Tanentzapf, 2010), cell apoptosis (Wei et al., 2020), and cell differentiation (Shen et al., 2019).

This review focuses on recent advances in bone and cartilage tissue engineering based on RGD-modified scaffolds. In addition to analyzing the possible influence of different RGD peptide sequence structure on bone and cartilage tissue engineering, we also deeply discussed mechanism of the biological effects of RGD peptide by the way of binding to different integrin receptors.

## Different Structures and Sequences of RGD

**FIGURE 1:**
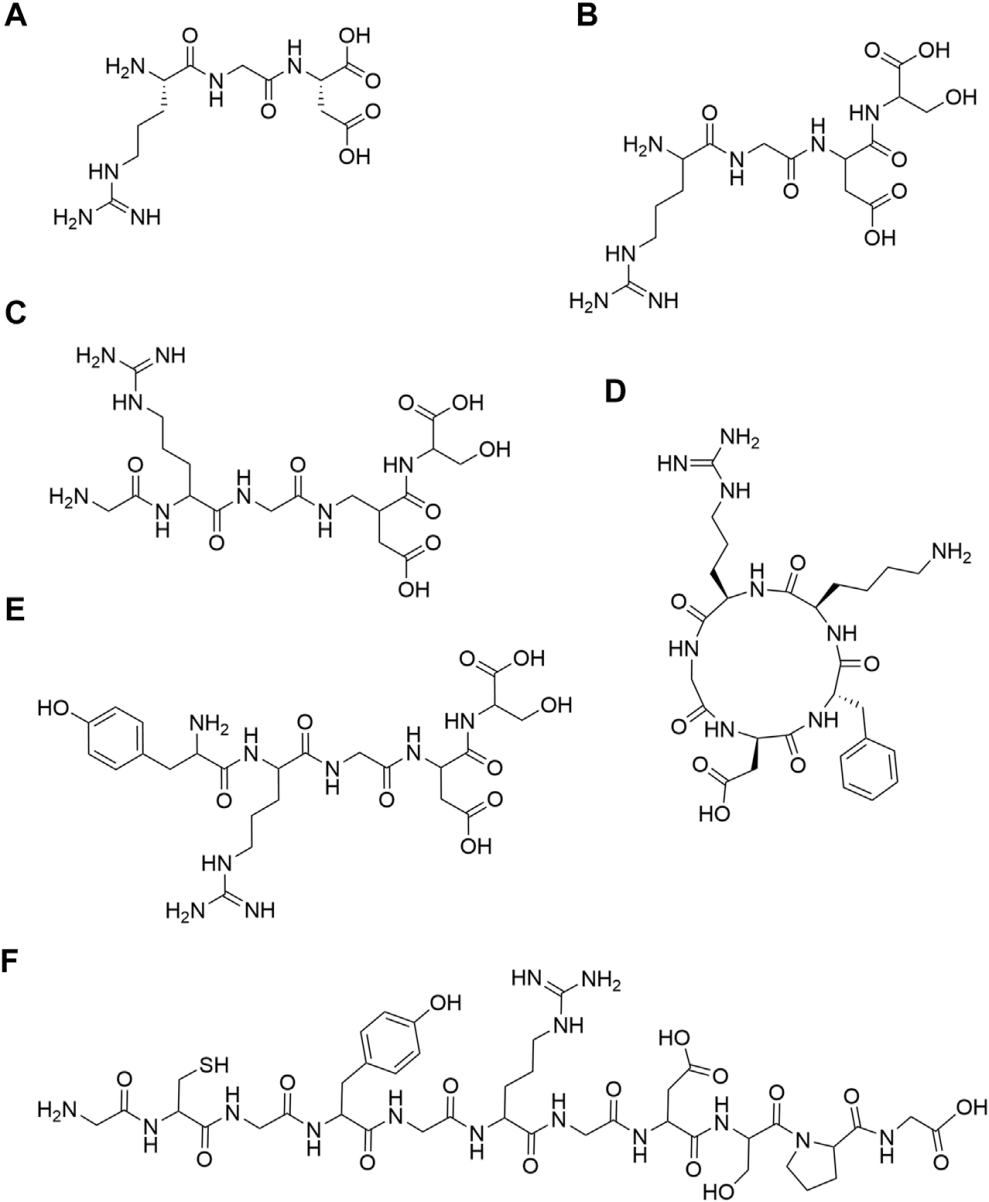
Chemical structures. **(A)** RGD; **(B)** RGDS; **(C)** GRGDS; **(D)** c (RGDfk); **(E)** YRGDS; **(F)** GCGYGRGDSPG.

RGD is a cell adhesion motif found in many ECM (Colombo and Bianchi, 2010). In 1984, Pierschbacher et al. first discovered the RGD peptide (Figure 1A) in fibronectin (Pierschbacher and Ruoslahti, 1984). Subsequently, it was found that RGD peptides were widely present in fibronectin, laminin, fibrinogen, osteopontin and vitronectin (Ruoslahti, 1996). RGD can be divided into RGD and RGD polypeptide. The former is a tripeptide sequence of RGD, and the latter is a functional peptide containing RGD. In the field of bone and cartilage tissue engineering, a variety of RGD-modified hydrogels with different structures have been used for bone and cartilage repair. In the aspect of bone repair, RGD structures that are widely used include RGDS (Benoit and Anseth, 2005) (Figure 1B), GRGDS (Paxton et al., 2009) (Figure 1C), c (RGDfk) (Tang et al., 2010; Bell et al., 2011; Peng et al., 2011; Jäger et al., 2013; Ye et al., 2015; Ye et al., 2016) (Figure 1D) and YRGDS (Burdick and Anseth, 2002; Yang et al., 2005; Steinmetz and Bryant, 2011; Reid et al., 2013) (Figure 1E). What’s more, RGD structures are widely used in cartilage repair, include c (RGDfk) (Cao et al., 2014; Li et al., 2015a; Li et al., 2015b) (Figure 1C), YRGDS (Bryant et al., 2008; Villanueva et al., 2009; Steinmetz and Bryant, 2011; Kim et al., 2015) (Figure 1E), RGDS (Salinas et al., 2007; Kloxin et al., 2009; Callahan et al., 2013a) (Figure 1B) and GCGYGRGDSPG (Kudva et al., 2017; Kudva et al., 2018a; Kudva et al., 2018b) (Figure 1F). The detailed structure diagram is shown in Figure 1. RGD peptides are mainly divided into linear and cyclic RGD peptides. Interestingly, cyclic RGD peptides are thought to be more active than linear RGD peptides. The probable reason is that cyclic peptides are more resistant to proteolysis and can bind to integrin receptors with a higher affinity (Verrier et al., 2002; Frochot et al., 2007). In addition, the study of Heller showed that cyclic RGD is more beneficial to bone repair *in vivo* than linear RGD (Heller et al., 2018).

## The Synthesis of RGD

Merrifield created and developed the method of solid phase peptide synthesis (SPPS), which greatly simplified the synthesis and purification of polypeptides, greatly improved the productivity, made the synthetic synthesis of various polypeptides feasible, and provided convenience for the modification of biological materials by polypeptides.

At present, there have been many reports on the synthesis methods of RGD peptide and its analogues, including enzyme-catalyzed synthesis, solid-phase synthesis and liquid-phase synthesis. Among them, Huang (Huang et al., 2005). reported the enzyme-catalyzed synthesis method, but the catalytic activity of the enzyme was affected by a variety of factors such as the reaction solvent system, the ratio of the dosage of the reaction substrate, reaction temperature, pH value, and reaction time. And the reaction conditions are strict and difficult to control. Kumagai et al. (1991) reported the liquid-phase synthesis method for RGD peptide, Liquid-phase synthesis is relatively simple, rapid and cost little, but due to the large pollution, complex reaction and other reasons, people prefer to use SPPS method. SPPS method to synthesis RGD peptide and its analogues has superiority of mild reaction conditions, simple reaction operation and easy automation, but it still has the disadvantages of high cost, low yield and not suitable for mass production (Sulyok et al., 2001; Kuijpers et al., 2007).

Cyclic RGD peptide is reported to be more stable due to rigidity of the ring and low degradability by enzyme (Bogdanowich-Knipp et al., 1999). However, inducing cyclic structure to RGD peptide brings additional challenge. In tradition strategy, cyclization was performed after cleavage the peptide from resin, using different coupling reagent/base system (Frochot et al., 2007) or just using NH_4_OH aqueous solution (Wang et al., 2005) to cyclization. Furthermore, in Wang’s work, the cyclization of pentapeptides was taken on the solid support, using benzotriazol-1-yl-oxy-tris-pyrrolidinophosphonium hexafluorophosphate (PyBOB), 1-hydroxybenzotriazole (HOBT) and N, N-Diisopropylethylamine (DIPEA) to from the cyclic peptide, while the side chain of Asp was conjugated to resin (Wang et al., 2005).

## The Active Site Integrin as the Mechanism of RGD Action

**FIGURE 2:**
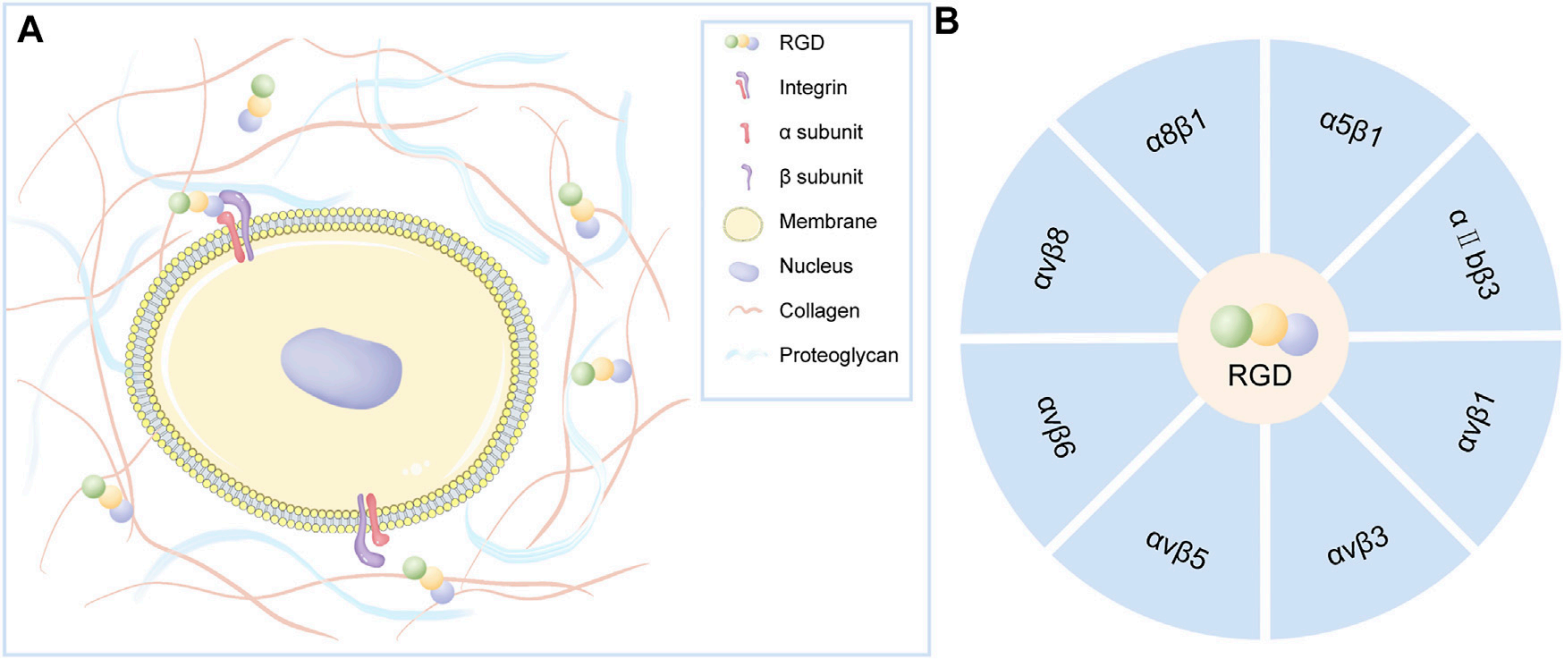
**(A)** Integrins on the cell membrane are composed of α and β subunits that act as transmembrane receptors mediating cell attachment to the extracellular matrix. Certain integrins can specifically recognize RGD polypeptides. **(B)** Integrin that specifically recognizes RGD polypeptides.

**FIGURE 3:**
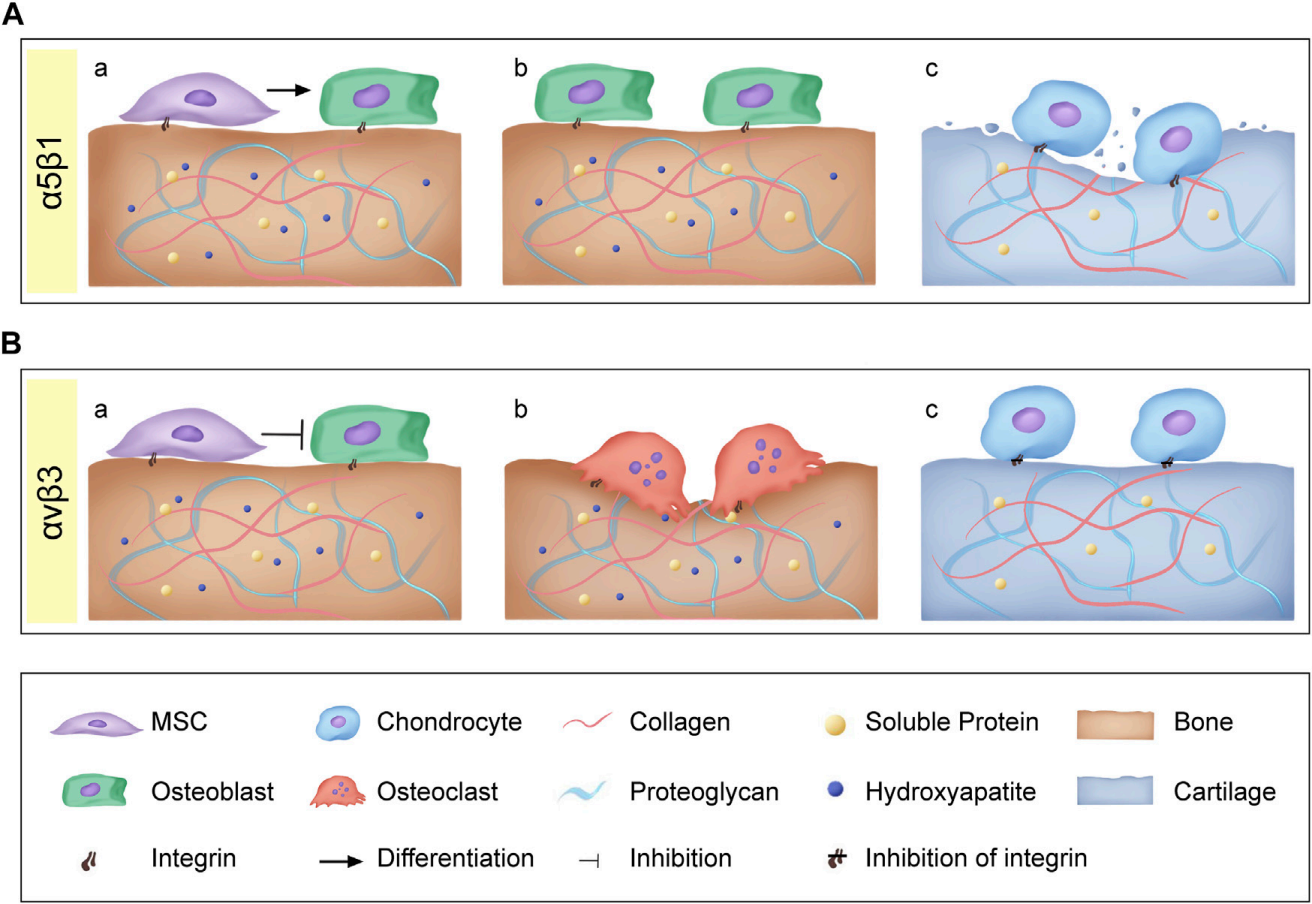
**(A)** Integrins α5β1: (a) α5β1 promote osteoblastic differentiation of mesenchymal stem cells; (b) α5β1 promote osteoblast proliferation and bone formation; (c) α5β1 also activates pro-inflammatory and catabolic responses leading to cartilage matrix degradation. **(B)** Integrins αvβ3: (a) αvβ3 Inhibit MSCs proliferation and osteogenic differentiation; (b) αvβ3 promote bone resorption; (c) Inhibition of αvβ3 could significantly inhibit osteoarthritis (OA) inflammation and decrease OA progression.

RGD is a specific ligand for integrins on cell membranes. Integrins on cell membranes are composed of α and β subunits, which are important transmembrane receptors that mediate the attachment of cells to extracellular matrix (Figure 2A). The combination of α and β subunits forms 24 kinds of integrins. Only some integrins recognize RGD sequences in natural ligands (Figure 2B), which are: α8β1, α5β1, αⅡbβ3, αvβ1, αvβ3, αvβ5, αvβ6, αvβ8 (Barczyk et al., 2010). Among them, α5β1 and αvβ3 integrins play a major role in bone and cartilage repair (Figure 3).

RGD can block α5β1 and prevent the maturation of bone nodules (Moursi et al., 1996). Integrin α5β1 helps to recruit Mesenchymal stem cells (MSCs) to the defect site for repair. Several studies have shown that α5β1 can promote the osteogenic differentiation of MSCs *in vitro* (Hamidouche et al., 2009; Martino et al., 2009). Studies have shown that αvβ3 seems to have an inhibitory effect in osteogenic differentiation (Cheng et al., 2001; Martino et al., 2009). Martino et al. found that blocking αvβ3 could promote the proliferation and osteogenic differentiation of MSCs (Martino et al., 2009). Similarly, the overexpression of αvβ3 can inhibit proliferation and the expression of osteogenic gene bone sialoprotein, ALP, and collagen I (Cheng et al., 2001).

Integrins can not only affect the differentiation of MSCs into osteoblasts, but also play an important role in bone formation and resorption. α5β1 integrins have been identified as essential for osteoblast survival and bone mineralization. Inhibiting the expression of α5β1 will lead to a low osteoblast survival rate (Dufour et al., 2008) and bone loss (Schneider et al., 2001; Dufour et al., 2008). Compared with the role of α5β1 in osteoblasts, αvβ3 is mainly closely related to osteoclasts. αvβ3 is an important adhesion integrin of osteoclasts, so inhibition of αvβ3 will lead to osteoclast apoptosis (Horton, 1997). In addition, studies have shown that through specific antagonism of αvβ3 integrin, it can inhibit bone resorption and increase bone mineral density (Horton, 2001; Cacciari and Spalluto, 2005).

RGD binding integrin is upregulated in osteoarthritic cartilage. A study has shown that the expression of integrins α5β1 gradually decreases during the differentiation of MSCs into cartilage (Goessler et al., 2008). Therefore, integrin α5β1 may affect undifferentiated MSCs, and with the progress of differentiation, it seems necessary to induce the phenotype of chondrocytes by reducing this receptor. Interestingly, Tao et al. found that by blocking α5β1 receptor can significantly reduce the enhancement of fibronectin (FN) on chondrogenic differentiation of chondrogenic progenitor cells (CPC) (Tao et al., 2018).

OA is one of the common bone and cartilage diseases, its pathological changes include cartilage erosion and loss on the joint surface (Pritzker et al., 2006). Fibronectin fragments are produced when the cartilage matrix is damaged. It’s binding with α5β1 can activate pro-inflammatory and catabolic responses, which will lead to cartilage matrix degradation (Loeser, 2014). Furthermore, many studies have shown that α5β1, as RGD binding integrin, is upregulated in osteoarthritic cartilage, promoting the expression of inflammatory signals, and ultimately accelerating the development of OA (Ostergaard et al., 1998; Attur et al., 2000; Almonte-Becerril et al., 2014; Candela et al., 2016). In addition to integrin α5β1, normal chondrocytes also express αvβ3, α1β1, αvβ5 and α3β1 (Woods et al., 1994). Among them, αvβ3 also plays a certain regulatory role in OA. Wang et al. shown that blocking αvβ3 can significantly inhibit the inflammation of OA and weaken the progression of OA (Wang et al., 2019). In addition, Mukundan et al. also confirmed that αvβ3 can reduce the production of inflammatory factors such as IL-1B, NO and PGE2, and negatively regulate the progression of OA (Attur et al., 2000).

## Application of RGD in Bone Tissue Engineering

RGD interacts with specific receptors on the surface of integrin and is therefore called a stimulant of cell adhesion. It is immobilized on the polymer surface to activate cell proliferation, regulate cell metabolism and extracellular matrix synthesis (de Jonge et al., 2008). RGD is often composed into PEG hydrogels to enhance cell viability. However, researchers usually focus on RGD’s ability to promote cell adhesion and proliferation. Whether RGD peptide can promote cell differentiation is still a controversial issue. In the following sections, we will discuss the effects of RGD peptides on cell differentiation in bone and cartilage tissue engineering.

The application scenarios of bone tissue engineering are mainly large-area bone defects, bone necrosis and bone nonunion caused by trauma (Kim et al., 2017). In the face of strong demand, supports represented by titanium alloy (Dard et al., 2000), PCL (Richbourg et al., 2019; Alipour et al., 2020), phosphate composites (Lin et al., 2019) and polyethylene glycol polymers (Wang et al., 2017) have been produced in the field of bone tissue engineering. These tissue engineering supports usually have high mechanical properties; whereas it’s accompanied by poor cell adhesion. RGD Peptides are often added to these scaffolds to improve their cell adhesion. The results shown that titanium alloy, PCL and phosphate complexes can significantly promote bone repair and healing after adding RGD Peptides (Lin et al., 2019; Alipour et al., 2020). Among them, many studies have shown that the surface modification of RGD by titanium alloy is beneficial to the early adhesion and spread of osteoblasts, and to the proliferation and differentiation of cells in the later stage (Ferris et al., 1999; Elmengaard et al., 2005; Heller et al., 2018).

PEG hydrogel also has the disadvantage of poor cell adhesion. While titanium alloy combined with RGD had achieved good results in the field of bone repair, studies of Jäger (Jäger et al., 2013), Benoit (Benoit and Anseth, 2005) and Bell (Bell et al., 2011) showed that RGD peptide combined with polyethylene glycol hydrogel did not promote stem cell osteogenesis. It even inhibits the osteogenic differentiation of cells. Tosatti (Tosatti et al., 2004) found that RGD-containing peptide GCRGYGRGDSPG reduced enhancement of osteoblast differentiation by poly-(_L_-lysine)-*graft*-PEG-coated titanium surfaces. The results of Bell (Bell et al., 2011) showed that RGD increased the number of cells, but decreased the markers of osteoblast differentiation. Moreover, Smith believed that in continuous gradient culture, low RGD concentrations were more conducive to osteogenic differentiation than high RGD concentrations (Callahan et al., 2013c).

There are also results showing that RGD peptide combined with PEG hydrogel can promote osteogenesis. Kim found that an injectable hydrogel based on MPEG (methoxy polyethylene glycol) -PCL-RGD could promote osteogenic differentiation of stem cells. Moreover, they suggested that focal adhesion kinase (FAK) protein kinase B (AKT) and FAK extracellular signal-regulated kinase (ERK) also played roles in osteogenic differentiation in the RGD-integrin-mediated pathway (Kim et al., 2020). Burdick (Burdick and Anseth, 2002) thought that compare with 0 mM, 0.5 mM RGD, 5 mM concentration of PEG-DA-RGD hydrogel had a more significant ability to promote cell mineralization. The results of Yang (Yang et al., 2005) showed that the expression of bone related markers Osteocalcin (OCN) and alkaline phosphatase (ALP) increased significantly with the increase of RGD concentration. Wong (Wong et al., 2017)’ data showed that high RGD tether mobility delayed the early adhesion and spreading of human mesenchymal stem cells (hMSCs), leading to compromised osteogenic differentiation at a later stage. In contrast, hMSCs cultured on substrate with restricted RGD tether mobility, achieved either via a shorter PEG linker or magnetic force, showed significantly better adhesion, spreading, and osteogenic differentiation. Moreover, PEG-RGD regulated the osteogenic differentiation of MSCs by changing the aspect ratio and shape of cells in 2D culture (Tang et al., 2010; Peng et al., 2011). Interestingly, the results of Steinmetz (Steinmetz and Bryant, 2011) showed that although simple RGD inhibited osteogenic differentiation, RGD could promote osteogenesis through dynamic compression of hydrogel scaffolds. In addition, Nam (Nam et al., 2019) found that faster relaxation of RGD functionalized alginate -PEG hydrogels enhanced osteogenic differentiation of MSCs.

In conclusion, there may be three reasons for the different results. First, the RGD adhesion peptide sequences used in each study are different. Some results show that cyclic RGD has better biological activity than linear RGD; Second, different cells adopt different integrin sites, which may activate different pathways and induce the opposite results; Third, there are different types of polyethylene glycol hydrogels. Their spatial structures are different, their effects on cells are different; and the time of degradation of hydrogels is also different. The suitable degradation time of tissue engineering scaffolds is very important. The slow degradation will prevent the growth of new bone, while the fast degradation will lead to the failure of new tissue to grow in time. The results of Thoma’s study showed that polyethylene glycol hydrogels with RGD had better degradability and improved bone formation (Thoma et al., 2011).

Beyond that, many studies have found that RGD polypeptide functionalized PEG-based hydrogels are very suitable scaffolds for bone tissue engineering (Nuttelman et al., 2005; Pan et al., 2014; Gao et al., 2015; Carles-Carner et al., 2018; Chahal et al., 2020). Although their results showed that the constructed hydrogel system had a significant osteogenic effect, RGD polypeptide in these hydrogel systems might mainly play the role of cell adhesion. They still lacked a control group to show that RGD promotes osteogenesis. The reasons for their conclusions are complex and most likely closely related to other components of the system that promote osteogenesis, such as: calcium phosphate composites (Chahal et al., 2020), hydroxyapatite nanoparticles (Pan et al., 2014; Carles-Carner et al., 2018), acrylated matrix metalloproteinase (MMP)-sensitive peptide (Gao et al., 2015), and ethylene glycol methacrylate phosphate (EGMP) (Nuttelman et al., 2005). It’s worth noting that nanoparticles modified with RGD peptides can be used to treat diseases (Liu et al., 2018; Deng et al., 2021) or deliver specific genes (Kong et al., 2015). Bone morphogenetic protein is an important growth factor for osteogenesis, GUAN et al. utilized PEG molecules and RGD peptide (thermo-activated thiol-yne and copper-free alkyne and azide click reactions) to achieve reverse gradients and create countercurrent distributions of fibroblast growth factor 2 (FGF-2) and bone morphogenetic protein 2 (BMP-2) gradients (Guan et al., 2016).

## Application of RGD in Cartilage Tissue Engineering

Once damaged, cartilage is difficult to repair due to the lack of nerves and blood vessels. There are also many studies on the treatment of cartilage defects with PEG combined with RGD tissue engineering scaffolds in recent years.

It is also controversial whether polyethylene glycol combined with RGD hydrogel promotes cell differentiation. Kudva’s research in recent years showed that 150 um RGD could promote human periosteal stem cells into cartilage (Kudva et al., 2018a). It is interesting to note the same RGD sequence structure and their research of human articular cartilage cells results showed that 150 μm RGD can promote cartilage cells *in vitro* plant regeneration (Kudva et al., 2017). It suggests different cells with the same RGD sequence will show different results of differentiation. Zhang’s results also showed that RGD polypeptides could improve the function of the cartilage cells, but would cause cartilage cell hypertrophy and slightly to differentiation tendency (Zhang et al., 2015). Li (Li et al., 2015a) found that RGD peptides nanoscale spatial arrangement of cartilage cells to differentiation may also be affected, and sparse RGD spatial arrangement could reduce cartilage cells to differentiation. Li’s results also showed that large RGD nano spacing could promote the differentiation of mesenchymal stem cells into cartilage (Li et al., 2015b). Moreover, the results illustrated that chondrocytes dedifferentiation were more likely to occur in the condition of larger sizes and higher aspect ratios (Cao et al., 2014).

Contrary to the results of Kim (Kim et al., 2015) and Vonwil (Vonwil et al., 2010), Smith believed that the cartilage phenotype and extracellular matrix secretion of human chondrocytes are inhibited with the increase of RGD concentration (Callahan et al., 2013b). Some scholars believed that appropriate RGD concentration, mechanical stimulation, physicochemical stimulation, or time stimulation was the key to promote the chondrogenic phenotype of cells. Liu (Liu et al., 2010) found that under different concentrations (0, 1 mM, 5 mM) of RGD peptide mixed polyethylene glycol hydrogel, the 1 mM RGD was most conducive to the formation of human mesenchymal stem cells *in vitro*. Mechanical stimulation may cause the reaction between RGD and cells. Without dynamic loading, RGD had a negative effect on chondrocyte phenotype. After dynamic compression, chondrocyte phenotype and proteoglycan synthesis increased with the increase of RGD concentration (Villanueva et al., 2009). Moreover, physicochemical properties may influence chondrogenic differentiation of cells and soft hydrogels are more conducive to chondrogenesis differentiation (Callahan et al., 2013a; Carrion et al., 2016). Another interesting phenomenon is the time response of RGD to cells. RGD promotes the survival of hMSC encapsulated in PEG gel, and can induce the early stage of cartilage formation. Its persistence would limit the complete differentiation of cells (Salinas and Anseth, 2008; Kloxin et al., 2009).

In conclusion, different RGD adhesion peptide sequences, spatial distribution of RGD polypeptide, cells, concentrations of RGD polypeptide, mechanical stimulation, and even time response all affect chondrogenic differentiation. More high-quality studies are needed to confirm this phenomenon.

## Conclusion

RGD is a cell adhesion sequence found in extracellular matrix. There are many kinds of structures, and different structures may play different roles. At present, RGD sequences that are widely used in the field of bone and cartilage repair include RGDS, GRGDS, c (RGDfk) and YRGDS. RGD, as a polypeptide sequence, can be synthesized in many ways, such as: enzyme-catalyzed synthesis, solid phase synthesis and liquid phase synthesis. They have their own advantages and disadvantages, and the common synthesis method is solid phase synthesis. Integrin seems to play an important role in the bone and cartilage repair, its one of the important mechanisms of the RGD polypeptides action. There are eight integrins that recognize RGD sequences in natural ligands. The α5β1 and αvβ3 integrins play the main roles. The role of integrin in bone and cartilage repair is complex. In general, α5β1 promotes osteogenic differentiation, osteoblast proliferation and bone formation of MSCs. α5β1 also promotes inflammation and decomposition, leading to cartilage matrix degradation. αvβ3 inhibited MSCs proliferation and osteogenic differentiation and promoted bone resorption. Finally, inhibition of αvβ3 significantly inhibited OA inflammation. At present, the application of RGD polypeptide in bone tissue engineering and cartilage tissue engineering is not in-depth enough, and it is still very controversial whether RGD polypeptide can promote osteogenesis or cartilage formation. The different results may be related to the structural sequence of RGD, concentration, spatial structure, time effect, mechanical stimulation, and distribution of integrins on different cells. In conclusion, the application of RGD in bone and cartilage tissue engineering needs further research, especially to explore its mechanism with integrin. In addition, the time responsiveness, mechanical responsiveness, and repair ability of RGD in complex environment *in vivo* also need further research.
